# Caregiving responsibility and psychological distress among community-dwelling cancer survivors in the United States

**DOI:** 10.1007/s00520-024-09133-7

**Published:** 2025-01-07

**Authors:** Asos Mahmood, Hyunmin Kim, Satish Kedia, Alexandria Boykins, Joy V. Goldsmith

**Affiliations:** 1 Center for Health System Improvement, College of Medicine, University of Tennessee Health Science Center, Memphis, TN USA; 2 Department of Medicine-General Internal Medicine, College of Medicine, The University of Tennessee Health Science Center, Memphis, TN USA; 3 School of Health Professions, The University of Southern Mississippi, Hattiesburg, MS USA; 4 Division of Social and Behavioral Sciences, School of Public Health, The University of Memphis, Memphis, TN USA; 5 College of Graduate Health Sciences, The University of Tennessee Health Science Center, Memphis, TN USA; 6 Department of Communication and Film, the University of Memphis, Memphis, TN USA

**Keywords:** Cancer survivors, Informal caregivers, Surviving Caregiver, Caregiving burden, PHQ-4, Psychological distress

## Abstract

**Purpose:**

There are over 18 million cancer survivors in the U.S., with a projected increase of 24.4% over the next decade. Currently, little is known about the relationship between a cancer survivor’s caregiving responsibility and their psychological distress. This study examines whether cancer survivors who assume the role of informal caregivers (surviving caregivers) experience greater psychological distress than cancer survivors without caregiving responsibilities.

**Methods:**

Data were drawn from the National Cancer Institute’s Health Information National Trends Survey (HINTS5, Cycles 1 through 4, 2017–2020). The analytical sample included 2,579 U.S. cancer survivors. Caregiving responsibility was self-reported, and psychological distress was assessed through the Patient Health Questionnaire-4 (PHQ-4). Accounting for the complex design features of HINTS and jackknife replicate weights, a multivariable multinomial logistic regression model was fit to compute adjusted odds ratios (aORs) and their associated 95% confidence intervals (CIs).

**Results:**

Overall, 19.3% of cancer survivors had mild psychological distress, and 10.9% had moderate to severe psychological distress. Approximately 19.1% of the cancer survivors self-reported caregiving responsibilities. Compared to cancer survivors with no caregiving responsibilities, surviving caregivers had more than twofold greater odds of experiencing mild (aOR = 2.25; 95% CI: 1.17, 4.29) and moderate to severe (aOR = 2.18; 95% CI: 1.07, 4.46) psychological distress. Other factors associated with greater psychological distress among cancer survivors included female sex, lower perceived health status, and having one or more chronic diseases.

**Conclusions:**

Our findings indicate that caregiving among cancer survivors has a substantial adverse impact on their mental and emotional well-being. Cancer surviving caregivers are a distinct subgroup that navigates both survivorship and caregiving burdens at the same time. There is a need to identify and develop tailored interventions, programs, and resources for this vulnerable group of cancer survivors.

**Supplementary Information:**

The online version contains supplementary material available at 10.1007/s00520-024-09133-7.

## Introduction

The number of cancer survivors in the United States (U.S.) is steadily increasing, now representing a significant portion of the population [1]. As of 2022, there were more than 18 million cancer survivors in the U.S., with a projected 24.4% increase, reaching 22.5 million over the next decade [1, 2]. All individuals diagnosed with cancer, whether currently undergoing treatment, having completed treatment, or cancer-free, are considered cancer survivors [2]. The National Coalition for Cancer Survivorship (NCCS) states that a person is considered a cancer survivor from the time of diagnosis through the remainder of life [3]. Currently, more than two-thirds of U.S. cancer survivors (approximately 12 million) are 65 years of age or older [1].

In general, cancer survivors experience various physical and mental health challenges post-diagnosis [4–7]. For example, they are at greater risk of experiencing symptoms of mental and emotional distress, developing depressive disorders, and even several years after initial diagnosis or cancer treatment [8–10]. The well-being of cancer survivors depends on factors such as demographics, physical and mental health, and existing support and resources; however, many experience a lower quality of life compared to their cancer-free counterparts [11–14]. Furthermore, social determinants of health (SDoH), such as educational and economic factors, housing and neighborhood conditions, and social connectedness, largely contribute to disparities in physical and mental health outcomes among cancer survivors [15]. Moreover, vulnerable populations, particularly rural populations, sexual and gender minorities, and minoritized racial and ethnic populations, often face greater challenges navigating cancer survivorship [16, 17]. In particular, people from racial and ethnic minority groups often face unequal access to early treatment and high-quality healthcare. These disparities can adversely impact their physical and mental health, as well as overall well-being [18–20].

### Caregiving in cancer survivorship

Informal caregivers are individuals who provide essential care and support to family and loved ones who need help taking care of themselves, and they have become an integral part of the healthcare system [21]. During 2021–2022, approximately 20.1% of the U.S. adult population self-identified as informal caregivers, with about one-third (35.4%) of them being aged 60 years and older [22]. Evidence suggests that greater caregiving responsibility, both in terms of frequency and intensity, is associated with increased psychological distress, including anxiety and depression [23]. Compared to younger caregivers, older caregivers are more likely to have poor health and a rapid decline in health due to increased caregiving responsibilities [24, 25]. Notably, anxiety and depression are common among caregivers of stroke, dementia, and cancer patients [26–30].

Cancer survivors who also have caregiving responsibilities (henceforth ‘surviving caregivers’) are a unique group of individuals who navigate survivorship and caregiving burdens simultaneously. The complex interplay of caregiving and navigating survivorship is likely to exacerbate the challenges and stressors already experienced by these individuals. Thus, cancer survivors who assume a “dual role” have poorer health outcomes compared to the general population on a range of health-related measures, including general physical and mental health, physical activity, and sleeping habits [31]. While significant research has distinctively examined the physical and mental health status among individuals with a history of cancer [1, 4, 32] and among caregivers in general [33–35], little research has focused on mental health and psychological distress among surviving caregivers. Specifically, much less is known about the dynamic relationships among cancer survivorship, informal caregiving responsibility, and psychological distress.

### Theoretical background

Cancer survivors experience long-term psychological, social, and physical effects related to cancer diagnosis and treatment. Survivors who also assume caregiving responsibilities are likely to experience compounded adverse outcomes by shouldering these two highly stressful roles simultaneously. Given the potentially complex interplay between illness survivorship status, caregiving demands, and overall health, we conceptualized our study within the context of the Stress Process [36] and the Life Stress [37] models to investigate these relationships. Pearlin et al.’s Stress Process Model (1981) helps examine how caregiving stressors interact with social and environmental factors to influence psychological distress. The model demonstrates two domains of stressors, primary (e.g., caregiving responsibilities) and secondary (e.g., socioeconomic status, role strain, etc.) [36]. Ensel & Lin’s (1991) Life Stress Model assumes a relationship between individual vulnerability, external stressors, and the onset of symptoms of psychological distress. Ensel and Lin focus on the ways life events and stressors (e.g., loss of a job, divorce, or, in this case, survivors assuming caregiving roles) influence psychological well-being [37]. These frameworks allow exploration of the key determinants that potentially exacerbate or mitigate surviving caregivers’ psychological distress because both frameworks emphasize that stress and the development of psychological distress are shaped by multiple factors.

In the present study, we explore whether surviving caregivers have increased psychological distress (symptoms of stress, anxiety, and depression) compared to cancer survivors with no caregiving responsibilities. We hypothesize that having caregiving responsibility is associated with an increased likelihood of psychological distress among cancer survivors. Investigating this association can support the development of tailored interventions or policies aimed at reducing psychological distress and enhancing mental health for surviving caregiver individuals. Furthermore, the study's findings are expected to contribute to the cancer survivorship and caregiving literature by assessing the unique needs and health-related concerns of this vulnerable group.

## Methods

### Data sources and study settings

This study utilized secondary data from four cycles of the fifth iteration of the Health Information National Trends Survey (HINTS5), fielded between 2017 and 2020. HINTS is administered by the National Cancer Institute (NCI) as a cross-sectional survey of noninstitutionalized U.S. adult populations (aged ≥ 18 years) [38–40]. Survey goals include collecting information on access to, need for, use of, and trends in health- and cancer-related information, as well as health- and cancer-related knowledge, perceptions, attitudes, and behaviors [38, 41–43]. Furthermore, HINTS is a valuable data resource for tracking and monitoring changes in health communication and digital health information technology and their impacts on health outcomes, healthcare quality, and health and healthcare disparities in the U.S. [41, 43]. The overall response rates for HINTS5, Cycles 1 through 4, were 32.4%, 32.9%, 30.3%, and 37.0%, respectively. Additional information about the survey and access to publicly available data can be found on its official website at https://hints.cancer.gov/.

### The study sample

Merging data from the four cycles of HINTS5 resulted in an overall sample of 16,092 respondents. The current study used the inclusion criteria of having been diagnosed with cancer. A personal history of cancer was assessed through the HINTS5 survey question “*Have you ever been diagnosed as having cancer?*” to which response options were “yes vs. no.” Thus, our population of interest in the current study and the analyzed sample included only survey participants with an affirmative response to this survey item and are referred to as cancer survivors throughout, resulting in a final sample size of 2,579 study participants. See e-Fig. 1 (supplemental file) for the study’s inclusion and exclusion criteria.

### Psychological distress

Cancer survivors’ psychological distress was assessed through the Patient Health Questionnaire-4 (PHQ-4) as the main dependent variable of interest in the current study. The PHQ-4 is a validated 4-item tool for anxiety and depression [44]. The tool is considered a relevant screening instrument with high sensitivity and specificity for mental and emotional health conditions [45–48]. Respondents were asked, “*Over the past 2 weeks, how often have you been bothered by any of the following problems?”*: 1) *“Little interest or pleasure in doing things”*; 2) *“Feeling down, depressed, or hopeless”*; 3) *“Feeling nervous, anxious, or on edge”*; and 4) *“Not being able to stop or control worrying*.” Response options were represented through a 4-point Likert-like scale including 1) nearly every day; 2) more than half the days; 3) several days; and 4) not at all. We reverse-scored (0 [not at all] to 3 [nearly every day]) and summed responses to generate the PHQ-4 score scale [46, 49]. Scores can range from 0–12, with higher scores indicating higher levels of psychological distress. Specifically, scores of 0–2 signify none or minimal, 3–5 mild, 6–8 moderate, and 9–12 severe psychological distress. For this study, we created a categorical variable for psychological distress with three sublevels of “none, mild, and moderate to severe” psychological distress. The subcategories “moderate” and “severe” were collapsed into one level because of the small cell size in the latter. This collapse aimed to increase the precision of the computed values and reduce variability in the analytical model.

### Caregiving responsibility

Caregiving responsibility was the independent variable of interest and was determined based on one survey item. Participants were asked, “*Are you currently caring for or making health care decisions for someone with a medical, behavioral, disability, or other condition?*” Respondents had the option of selecting “yes” and marking caregiving responsibility for all those that apply from a list of care recipients: “*a child/children, a spouse/partner, a parent/parents, another family member, a friend or other non-relative*.” Or had the option of “no,” if they did not have any caregiving responsibilities. Thus, informal caregiving responsibility in the current study is represented through a binary variable, “yes” vs. “no.”

### Study covariates

Based on existing literature and theoretical frameworks, we included data on several cancer survivors' sociodemographics, healthcare access, health status, and health behaviors. Age was defined as “ < 65 years vs. ≥ 65 years.” Self-reported sex was reported as “male vs. female,” and race and ethnicity were categorized into “non-Hispanic White, non-Hispanic Black, Hispanic, and non-Hispanic Asian and others.” Marital status was grouped into “married or living as married,” “divorced, widowed, or separated,” and “single or never married.” Residential location was defined based on the metropolitan statistical area (MSA) where respondents were located at the time of the survey, “MSA vs. non-MSA.” Respondents’ educational attainment was categorized into “less than high school, high school graduate, some college, and college graduate or more,” and annual household income (US$) as “ < 20,000, 20,000—34,999, 35,000—49,999, 50,000—74,999, and ≥ 75,000.” Healthcare access was evaluated by whether participants had health insurance coverage, “yes vs. no,” and a regular healthcare provider, “yes vs. no.” Self-rated general health status was assessed on three levels, “excellent or very good, good, and fair or poor.” The number of other diagnosed chronic medical conditions (CCs) was dichotomized into “none vs. one or more,” and history of cancer was reported as “ < 5 years and ≥ 5 years.” In addition, measures of respondents’ body mass index (BMI [kg/m^2^]), “underweight or normal (≤ 24.9), overweight (25—29.9), obese (≥ 30),” and their smoking status, “current, former, and never,” were incorporated as covariates.

### Statistical analysis

The analyses for this study were initiated by cleaning the data and exploring data missingness and patterns within the missing data. Using the MI procedure in the SAS software program, we found no missingness patterns, and a few instances of missing observations were completely random. Subsequent steps of data analyses accounted for final person weights and jackknife replicate weights, as provided with the HINTS dataset, to produce population-level estimates and generate more accurate and robust standard errors of estimates. Unweighted frequencies and weighted proportions for all cancer survivor characteristics were calculated in one-way analyses. We also calculated frequencies and weighted percentages for cross-tabulated associations between cancer survivor characteristics and psychological distress status and used the Wald Chi-square test to check the significance of associations.

Finally, a multivariable multinomial logistic regression model was fitted (employing a generalized logit function) to evaluate associations between cancer survivors’ caregiving status and levels of psychological distress. Through the same model, we also explored the adjusted associations between other characteristics and levels of psychological distress among the general cancer survivor population. The final results included adjusted odds ratios (aORs) and their associated 95% confidence intervals (CIs). A multicollinearity test was performed during multivariable model fitting, and no threats of multicollinearity were found. Sensitivity analyses were performed to check for the robustness and validity of the findings (see e-Methods, Supplemental file). All analyses were conducted in the SAS statistical software program (version 9.4; SAS Institute Inc., Cary, NC), and two-tailed *P* values ≤ 0.05 were considered statistically significant.

The current study was drafted and reported in accordance with the Strengthening the Reporting of Observational Studies in Epidemiology (STROBE) statement [50]. Since the utilized secondary data were de-identified and publicly available, the study protocol did not require additional Institutional Review Board (IRB) approval [51].

## Results

Our analytical sample included 2,579 respondents, representing the U.S. national-level noninstitutionalized cancer survivor population across 4 years of HINTS5 data collection cycles. Approximately 19% of the cancer survivors experienced mild psychological distress, and close to 11% experienced moderate to severe distress. Nearly one-fifth (19.1%) reported caregiving responsibilities. Over half (50.5%) of the cancer survivors were 65 years of age or older. Cancer survivors were mainly females (57.1%), non-Hispanic Whites (79.3%), married or living as married (61.7%), and residing in an MSA in the U.S. (84.3%). Those with some college education or above accounted for 66%, and 53% had an annual household income of US$ ≥ 50,000. Cancer survivors with health insurance coverage and a regular healthcare provider were 96.4% and 84.0%, respectively. Approximately 38.2% reported being in excellent or very good health. More than two-thirds reported having one or more CC other than cancer (71.3%), and 70.4% had lived with a cancer diagnosis for 5 years or more. The cancer survivors reported being mainly overweight or obese (68.2%) and former or never smokers (88.2%) (see Table 1).

**Table 1:** Sociodemographic and other health or personal characteristics of cancer survivors (HINTS5, Cycles 1 through 4, 2017–2020, the United States)

	Sample (*n* = 2,579)
Characteristics	Frequency (weighted %)*
Psychological distress	
Normal	1,797 (69.8)
Mild	438 (19.3)
Moderate to severe	252 (10.9)
Caregiver	
Yes	377 (19.1)
No	2,131 (80.9)
Age	
< 65 years	927 (49.5)
≥ 65 years	1,597 (50.5)
Sex	
Male	999 (42.9)
Female	1,349 (57.1)
Race and ethnicity	
Non-Hispanic White	1,759 (79.3)
Non-Hispanic Black	224 (8.4)
Hispanic	184 (8.6)
Non-Hispanic Asian and others	114 (3.7)
Marital status	
Married or living as married	1,310 (61.7)
Divorced, widowed, or separated	966 (24.0)
Single or never married	249 (14.3)
Residential location	
MSA	2,239 (84.3)
Non-MSA	340 (15.7)
Education	
Less than high school	152 (6.8)
High school graduate	535 (27.2)
Some college	780 (37.8)
College graduate or more	1,058 (28.2)
Annual household income (US $)	
< 20,000	475 (17.2)
20,000–34,999	399 (14.3)
35,000–49,999	364 (15.5)
50,000–74,999	462 (18.9)
≥ 75,000	840 (34.1)
Health insurance coverage	
Yes	2,477 (96.4)
No	54 (3.6)
Have a regular healthcare provider	
Yes	2,145 (84.0)
No	382 (16.0)
Self-rated general health status	
Excellent or very good	999 (38.2)
Good	915 (36.4)
Fair or poor	630 (25.4)
CCs	
None	623 (28.7)
One or more	1,956 (71.3)
Years with cancer	
< 5 years	699 (29.6)
≥ 5 years	1,723 (70.4)
BMI (kg/m^2^)	
Underweight or normal (≤ 24.9)	799 (31.8)
Overweight (25–29.9)	885 (36.2)
Obese (≥ 30)	828 (32.0)
Smoking status	
Current	272 (11.8)
Former	924 (34.0)
Never	1,360 (54.2)

MSA, Metropolitan Statistical Area; US, United States; CC, Chronic Conditions; BMI, Body Mass Index (calculated as weight in kilograms divided by height in meters squared)

^*^Frequencies represent sample frequencies; proportions are population-level estimates that were generated by adjusting for complex survey features of the HINTS data (N = 91.1 million)

Surviving caregivers represented a higher proportion of all cancer survivors experiencing mild (28.3%) or moderate to severe (31.3%) psychological distress, compared to the proportion of surviving caregivers experiencing normal distress levels (14.4%) (see Table 2). Psychological distress, in general, was greater among females, survivors aged < 65 years, those with lower annual household income and educational attainment, survivors in ‘fair or poor’ general health, and those with one or more CC (see Table 2).

**Table 2:** Cross-tabulated associations between psychological distress and other individual characteristics of cancer survivors (n = 2,579, HINTS5, Cycles 1 through 4, 2017–2020, the United States)

	Psychological Distress			
	Normal (n = 1797)	Mild (n = 438)	Moderate to severe (n = 252)	
Characteristics	Frequency (weighted %)^a^	Frequency (weighted %)^a^	Frequency (weighted %)^a^	*P*^b^
Caregiver				
Yes	213 (14.4)	89 (28.3)	64 (31.3)	< .001
No	1,548 (85.6)	333 (71.7)	179 (68.7)	
Age				
< 65 years	601 (46.6)	176 (54.7)	130 (63.4)	.001
≥ 65 years	1,164 (53.4)	255 (45.3)	117 (36.6)	
Sex				
Male	753 (47.7)	154 (34.8)	71 (30.3)	< .001
Female	888 (52.3)	243 (65.2)	162 (69.7)	
Race and ethnicity				
Non-Hispanic White	1,285 (81.5)	277 (73.4)	154 (76.0)	.10
Non-Hispanic Black	145 (6.7)	45 (14.5)	24 (8.6)	
Hispanic	106 (7.5)	42 (10.2)	27 (11.8)	
Non-Hispanic Asian and others	81 (4.3)	16 (1.9)	15 (3.6)	
Marital status				
Married or living as married	989 (65.9)	190 (51.9)	105 (56.9)	.028
Divorced, widowed, or separated	621 (22.5)	184 (25.1)	109 (26.6)	
Single or never married	159 (11.6)	48 (23.0)	32 (16.5)	
Residential location				
MSA	1,575 (85.3)	371 (81.9)	211 (81.3)	.32
Non-MSA	222 (14.7)	67 (18.1)	41 (18.7)	
Education				
Less than high school	60 (3.2)	48 (15.0)	31 (15.2)	< .001
High school graduate	342 (25.3)	98 (30.9)	64 (26.1)	
Some college	540 (38.8)	140 (36.1)	77 (39.3)	
College graduate or more	826 (32.7)	138 (18.0)	73 (19.4)	
Annual household income (US $)				
< 20,000	225 (12.0)	122 (24.5)	98 (33.4)	< .001
20,000–34,999	263 (13.7)	73 (14.1)	46 (17.7)	
35,000–49,999	262 (14.4)	65 (20.5)	26 (13.5)	
50,000–74,999	349 (20.2)	71 (18.9)	32 (12.8)	
≥ 75,000	672 (39.7)	99 (22.0)	47 (22.6)	
Health insurance coverage				
Yes	1,736 (97.3)	417 (95.5)	237 (92.2)	.15
No	23 (2.7)	15 (4.5)	15 (7.8)	
Have a regular healthcare provider				
Yes	1,509 (84.3)	365 (86.3)	199 (79.5)	.33
No	251 (15.7)	63 (13.7)	51 (20.5)	
Self-rated general health status				
Excellent or very good	842 (46.9)	102 (23.0)	33 (14.9)	< .001
Good	634 (36.6)	171 (40.3)	74 (26.1)	
Fair or poor	299 (16.5)	159 (36.7)	141 (59.0)	
CCs				
None	504 (32.7)	68 (24.6)	21 (7.8)	< .001
One or more	1,293 (67.3)	370 (75.4)	231 (92.2)	
Years with cancer				
< 5 years	476 (28.9)	139 (33.0)	61 (26.3)	.43
≥ 5 years	1,228 (71.1)	174 (67.0)	176 (73.7)	
BMI (kg/m^2^)				
Underweight or normal (≤ 24.9)	572 (31.6)	124 (32.2)	70 (31.4)	.04
Overweight (25–29.9)	631 (37.7)	162 (37.9)	71 (25.1)	
Obese (≥ 30)	560 (30.7)	137 (29.9)	103 (43.5)	
Smoking status				
Current	143 (8.8)	53 (14.3)	61 (24.3)	.002
Former	648 (34.2)	163 (34.9)	84 (33.1)	
Never	993 (57.0)	217 (50.8)	103 (42.6)	

MSA, Metropolitan Statistical Area; US, United States; CC, Chronic Conditions; BMI, Body Mass Index (calculated as weight in kilograms divided by height in meters squared)

^a^ Frequencies represent sample frequencies; proportions are population-level estimates that were generated by adjusting for complex survey features of the HINTS data

^b^ Wald χ^2^ test

Table 3 presents the results of the multivariable multinomial regression model. Compared to cancer survivors with no caregiving responsibilities, surviving caregivers had approximately twofold greater odds of experiencing mild (aOR = 2.25; 95% CI: 1.17, 4.29) and moderate to severe (aOR = 2.18; 95% CI: 1.07, 4.46) psychological distress as opposed to having a normal psychological evaluation. Furthermore, in the adjusted model, we found a few other cancer survivor characteristics that were significantly associated with psychological distress. For example, compared to cancer survivors aged < 65 years, those aged 65 years and older had significantly lower odds of experiencing mild (aOR = 0.50; 95% CI: 0.31, 0.79) and moderate to severe (aOR = 0.31; 95% CI: 0.15, 0.61) psychological distress. Compared to males, female cancer survivors were more likely to experience mild (aOR = 1.56; 95% CI: 1.02, 2.41) and moderate to severe (aOR = 1.87; 95% CI: 1.02, 3.43) distress. Cancer survivors with some college degree (aOR = 0.31; 95% CI: 0.10, 0.97) and those with a college degree or more (aOR = 0.27; 95% CI: 0.10, 0.82) had significantly lower odds of mild psychological distress when compared to their counterparts with less than a high school education (see Table 3).

**Table 3:** Multivariable multinomial logistic regression results for associations between caregiving responsibility and levels of psychological distress among cancer survivors (n = 2,579, HINTS5, Cycles 1 through 4, 2017–2020, the United States)

	Psychological distress	
	Mild vs. Normal	Moderate to severe vs. Normal
Characteristics	Adjusted OR (95% CI)	Adjusted OR (95% CI)
Caregiver (ref: no)		
Yes	2.25 (1.17, 4.29)^a^	2.18 (1.07, 4.46)^a^
Age (ref: < 65 years)		
≥ 65 years	0.50 (0.31, 0.79)^b^	0.31 (0.15, 0.61)^c^
Sex (ref: male)		
Female	1.56 (1.02, 2.41)^a^	1.87 (1.02, 3.43)^a^
Race and ethnicity (ref: non-Hispanic white)		
Non-Hispanic Black	0.77 (0.36, 1.67)	0.49 (0.20, 1.16)
Hispanic	0.87 (0.32, 2.40)	0.68 (0.20, 2.37)
Non-Hispanic Asian and others	0.26 (0.10, 1.08)	0.48 (0.15, 1.59)
Marital status (ref: single or never married)		
Married or living as married	0.63 (0.29, 1.36)	1.02 (0.42, 2.50)
Divorced, widowed, or separated	0.71 (0.34, 1.48)	0.75 (0.30, 1.93)
Residential location (ref: MSA)		
Non-MSA	1.09 (0.56, 2.13)	0.93 (0.45, 1.92)
Education (ref: less than high school)		
High school graduate	0.41 (0.14, 1.21)	0.30 (0.10, 1.27)
Some college	0.31 (0.10, 0.97)^a^	0.43 (0.12, 1.59)
College graduate or more	0.27 (0.10, 0.82)^a^	0.45 (0.11, 1.82)
Annual household income (US $) (ref: < 20,000)		
20,000–34,999	0.71 (0.31, 1.63)	0.64 (0.27, 1.54)
35,000–49,999	0.60 (0.22, 1.64)	0.46 (0.18, 1.17)
50,000–74,999	0.80 (0.31, 2.05)	0.39 (0.16, 0.99)^a^
≥ 75,000	0.62 (0.24, 1.57)	0.33 (0.12, 0.86)^a^
Health insurance coverage (ref: no)		
Yes	2.08 (0.49, 8.92)	0.78 (0.14, 4.27)
Have a regular healthcare provider (ref: no)		
Yes	1.06 (0.49, 2.29)	0.61 (0.27, 1.37)
Self-rated general health status (ref: excellent or very good)		
Good	2.32 (1.32, 4.08)^b^	2.22 (1.05, 4.70)^a^
Fair or poor	4.27 (2.30, 7.92)^c^	10.81 (5.03, 23.23)^c^
CCs (ref: none)		
One or more	1.76 (0.93, 3.31)	6.12 (1.94, 19.31)^b^
Years with cancer (ref: < 5 years)		
≥ 5 years	0.93 (0.56, 1.55)	1.54 (0.81, 2.90)
BMI (kg/m^2^) (ref: underweight or normal [≤ 24.9])		
Overweight (25–29.9)	1.04 (0.60, 1.80)	0.61 (0.31, 1.17)
Obese (≥ 30)	0.75 (0.42, 1.36)	0.89 (0.48, 1.64)
Smoking status (ref: current)		
Former	1.20 (0.45, 3.18)	0.61 (0.24, 1.55)
Never	1.16 (0.47, 2.88)	0.47 (0.20, 1.11)

OR, Odds Ratio; CI, Confidence Interval; MSA, Metropolitan Statistical Area; US, United States; CC, Chronic Conditions; BMI, Body Mass Index (calculated as weight in kilograms divided by height in meters squared)

a) *P* < .05; b) *P* < .01; c) *P* < .001

Moreover, compared to cancer survivors with US$ < 20,000 in annual household income, those who reported an income of US$50,000–74,999 (aOR = 0.39; 95% CI: 0.16, 0.99) and US$ ≥ 75,000 (aOR = 0.33; 0.12, 0.86) had significantly lower odds of moderate to severe distress. Compared to cancer survivors who perceived their general health as excellent or very good, those who considered it good had higher odds of mild (aOR = 2.32; 95% CI: 1.32, 4.08) and moderate to severe (aOR = 2.22; 95% CI: 1.05, 4.70) distress. Similarly, those who perceived their health to be in fair or poor condition (vs. excellent or very good) had significantly higher odds of mild (aOR = 4.27; 95% CI: 2.30, 7.92) and moderate to severe (aOR = 10.81; 95% CI: 5.03, 23.23) psychological distress. Finally, compared to those with no reported CCs, those with one or more CCs had greater odds of moderate to severe psychological distress (aOR = 6.12; 95% CI: 1.94, 19.31) (see Table 3). Results from our sensitivity analyses confirmed the robustness of the adjusted findings as the direction, magnitude, and significance of associations did not substantially change (see e-Table 1, Supplemental file).

## Discussion

This study investigated associations between informal caregiving responsibilities and psychological distress among cancer survivors. Drawing upon a nationally representative sample of cancer survivors in the U.S., we found that caregiving responsibility was markedly associated with adverse mental and emotional health among surviving caregivers. These findings contribute to the literature by exploring mental health in the often-overlooked population of cancer surviving caregivers. Given that even cancer survivors without caregiving responsibilities have special medical, psychological, and social needs that require increased attention [1], surviving caregivers are more vulnerable to increased emotional, physical, and financial distress [52, 53]. Our findings indicate that caregiving exacerbates the negative mental and emotional impacts already experienced by cancer survivors. The findings suggest that the additional responsibility of caregiving offers a potentially plausible explanation for the increased psychological distress and poorer self-reported mental health experienced by surviving caregivers compared to cancer survivors with no caregiving role.

The present study also indicates that older age and higher education and income levels are associated with lower odds of psychological distress. However, factors such as female sex, lower perceived health status, and comorbid chronic disease are linked to greater psychological distress within the broader population of cancer survivors. Older cancer survivors are less likely to experience psychological distress compared to younger survivors (aged < 65), which is consistent with prior research findings that older cancer survivors have lower levels of psychological distress and depressive symptoms compared to younger cancer survivors [54, 55]. In this regard, perceived responsibilities associated with work and young children may offer a likely explanation for differences in self-reported psychological distress among younger cancer survivors.

Moreover, the results revealed several other sociodemographic factors associated with psychological distress among cancer survivors. While our findings indicate that psychological distress is more common among female cancer survivors, beyond cancer survivorship, there is a greater prevalence of psychological distress among women than men [56, 57]. The gender difference in reported psychological distress among cancer survivors may be attributable to perceived social support, dealing with the short- and long-term health effects of cancer, coping strategies, and other factors, such as fewer “protective” resources [58]. Furthermore, harmful stereotypes regarding masculinity and the consequent tendency for males not to report experiencing or seeking help for mental health concerns could be another reason for the higher prevalence of psychological distress among female survivors compared to males.

Additionally, our findings indicate that having a college degree or higher and an annual income over US$50,000 were protective against psychological distress. Scholarly evidence supports this finding, indicating the protective benefits of higher socioeconomic status (SES) on mental health [59, 60]. Given that SES, or its various proxies, can mediate or exacerbate distress, the findings align with existing studies that demonstrate the effects as a stressor or positive resource [36, 37, 59, 60]. For instance, Baum and colleagues observed that lower SES is frequently associated with higher levels of distress, an increased prevalence of mental health issues, and engagement in health-risk behaviors linked to stress [59]. Moreover, cancer survivors are increasingly likely to experience financial toxicity [61], a term that describes the financial burden caused by cancer care. Costs associated with cancer care include out-of-pocket costs for medications, transportation, and lost wages [62]. Cancer survivorship is an enduring experience, with many cancer survivors experiencing persistent financial burdens even after treatment ends due to ongoing medical costs and long-term disability or illness. Particularly, surviving caregiver groups with lower SES could be more vulnerable to experiencing the compounded economic burden related to the financial strain of caregiving and the financial toxicity of cancer survivorship.

Finally, our results showed that cancer survivors who perceived themselves to have fair or poor health or had one or more CC were more likely to experience mild and moderate to severe psychological distress. Prior studies have shown that poorly perceived health and comorbid medical conditions are correlated with experiencing symptoms of psychological distress [20, 63, 64]. Similarly, others indicate that reporting a worse quality of life or poor emotional health is simultaneously associated with increased numbers of CCs at cancer diagnosis [63]. According to Arneja and Brooks (2021), since CCs worsen in severity and complexity over time, comorbidities are likely to reduce cancer survivors' quality of life and emotional well-being. Furthermore, Tsai et al. (2023) reported that cancer survivors with one to two CCs were more likely to report several days of poor mental health, whereas survivors with 3 or more CCs were more likely to experience both frequent poor mental and physical health [20]. These prior and current study findings suggest that having a greater number of CCs beyond cancer could intensify the emotional burden on survivors. Future research may explore the deterrent and exacerbating factors on the path linking the number and types of CCs and psychological distress among cancer survivors.

The findings collectively underscore the relevance of the Stress Process and Life Stress models in elucidating the psychological distress encountered by cancer survivors, especially surviving caregivers. The Stress Process Model highlights the dual challenges that surviving caregivers encounter, managing primary stressors related to caregiving in conjunction with secondary stressors, including socioeconomic difficulties. This corresponds with the heightened psychological distress noted in cancer survivors, particularly among females, younger survivors, and individuals with lower SES. The Life Stress Model explicates the role of external stressors, including comorbid CCs and perceived poor health, in intensifying distress. Survivors who reported poor health or multiple CCs exhibited significantly elevated levels of distress, reinforcing the model's focus on individual vulnerability and stress. Both frameworks demonstrate the cumulative impact of caregiving alongside negative health and socioeconomic conditions, indicating that interventions must tackle both the immediate challenges of caregiving and the wider contextual factors to alleviate distress.

### Strengths and limitations

The current study is novel in its examination of surviving caregivers, bearing the burden of their own health coupled with care for others. Our study boasts several strengths, such as the use of a nationally representative sample of surviving caregivers—a vulnerable subgroup of cancer survivors—with special health-related needs. In this study, we also considered a variety of potential confounding factors as control measures. To ensure the interpretability of our results at the population level, we accounted for survey weights and jackknife replicate weights in the analyses. However, some limitations warrant consideration for future research. First, the study relied on self-reported information, raising the possibility of personal bias, which is also a risk due to the cross-sectional design of the HINTS. Second, we were unable to infer causality from the computed findings due to the cross-sectional nature of the data. Furthermore, the HINTS5 questionnaire shortfalls include not being able to capture the stage of care, remission, or type of cancer, nor being able to determine whether the sample respondents are currently receiving cancer treatment, or whether they were experiencing any late/lingering side effects from treatment, specifically for individuals considered to have no evidence of disease (NED), in remission, or cancer-free. Other limitations of the data include the inability to incorporate employment status, patient activation or coping style measures, contextual factors such as social support, or the level of caregiving burden/responsibility, potentially influencing psychological distress among surviving caregivers. Lastly, despite adjusting for the presence of chronic conditions, we could not control for their severity or duration. Therefore, we were bound by the availability of information in the HINTS dataset. These limitations present opportunities for future research.

## Conclusions

The findings here demonstrate the complex relationships among cancer survivorship, informal caregiving responsibility, and psychological distress, as evidenced by the increased odds of psychological distress among surviving caregivers compared to cancer survivors with no caregiving responsibilities. Furthermore, social determinants such as income and education, as well as age and CCs, were associated with the likelihood of psychological distress among cancer survivors. Future research should explore the health, well-being, and psychosocial needs of surviving caregivers. Moreover, future prospective studies should focus on mitigating the negative impacts of caregiving among cancer survivors, tailoring intervention programs, and providing resources to alleviate the strain on surviving caregivers. Additionally, it is critical to explore disparities in physical and mental health among various groups of surviving caregivers, particularly racial and ethnic minorities, who already experience worse health outcomes and self-reported health compared to their White counterparts. Our results further highlight the need for future qualitative or mixed-methods research that explores the experiences of surviving caregivers and their perceptions of appropriate supportive care services and resources.

Surviving caregivers need targeted policies and support programs tailored to their specific needs. Some professional organizations have endorsed screening all cancer patients and survivors for psychosocial distress [65–67]. The capacity of healthcare professionals to provide meaningful interventions for surviving caregivers and their care recipients may be improved by the development of a screening tool that is attentive to psychological distress, caregiver burden, and poor health outcomes. Because surviving caregivers face unique challenges, there is a need for increased access to and awareness of counseling services and tangible resources that enable them to navigate their dual roles while protecting their own physical, psychological, and emotional health. With the changing landscape of informal caregivers (i.e., aging caregivers with comorbidities and poor physical health) coupled with increases in cancer survivorship, the number of surviving caregivers will likely grow exponentially in the coming years. Further exploration of this issue will enable the development of more effective policies and initiatives to improve the quality of life for these individuals as they continue to navigate cancer survivorship along with their essential role of caregiving.

## Supplementary Information

Below is the link to the electronic supplementary material.

Supplementary file1 (PDF 165 KB)

## Data Availability

The data analyzed for the current study were obtained from the National Cancer Institute’s Health Information National Trends Survey; publicly available to access and download at https://hints.cancer.gov/
