# Supplementary material for: Caregiving responsibility and psychological distress among community-dwelling cancer survivors in the United States
Source: Support Care Cancer. 2025 Jan 7;33(2):75. doi: 10.1007/s00520-024-09133-7 (PMC11706889; doi:10.1007/s00520-024-09133-7)
Supplement: Supplementary file 1 — Supplementary file1 (PDF 165 KB) [file 520_2024_9133_MOESM1_ESM.pdf]

Title: Caregiving responsibility and psychological distress among community-dwelling cancer survivors in the United States

Authors: Asos Mahmood[1,2] · Hyunmin Kim[3] · Satish Kedia[4] · Alexandria Boykins[5] · Joy V. Goldsmith[6] ·

[1]Center for Health System Improvement, College of Medicine, University of Tennessee Health Science Center, Memphis, TN, USA

[2]Department of Medicine-General Internal Medicine, College of Medicine, The University of Tennessee Health Science Center, Memphis, TN, USA

[3]School of Health Professions, The University of Southern Mississippi, Hattiesburg, Mississippi, USA

[4]Division of Social and Behavioral Sciences, School of Public Health, The University of Memphis, Memphis, TN, USA

[5]College of Graduate Health Sciences, The University of Tennessee Health Science Center, Memphis, TN, USA

[6]Department of Communication and Film, the University of Memphis, Memphis, TN, USA

Corresponding Author:

Asos Mahmood, MD, PhD, MPH

Assistant Professor

Center for Health System Improvement – College of Medicine

The University of Tennessee Health Science Center

Email: amahmoo5@uthsc.edu

Supplemental Content

e-Figure 1. A flowchart depicting the study's inclusion and exclusion criteria.

e-Methods. Sensitivity analysis.

e-Table 1. Findings of the sensitivity analyses performed for the associations between caregiving responsibility and levels of psychological distress among cancer survivors (HINTS5, Cycles 1 through 4, 2017 – 2020, the United States).

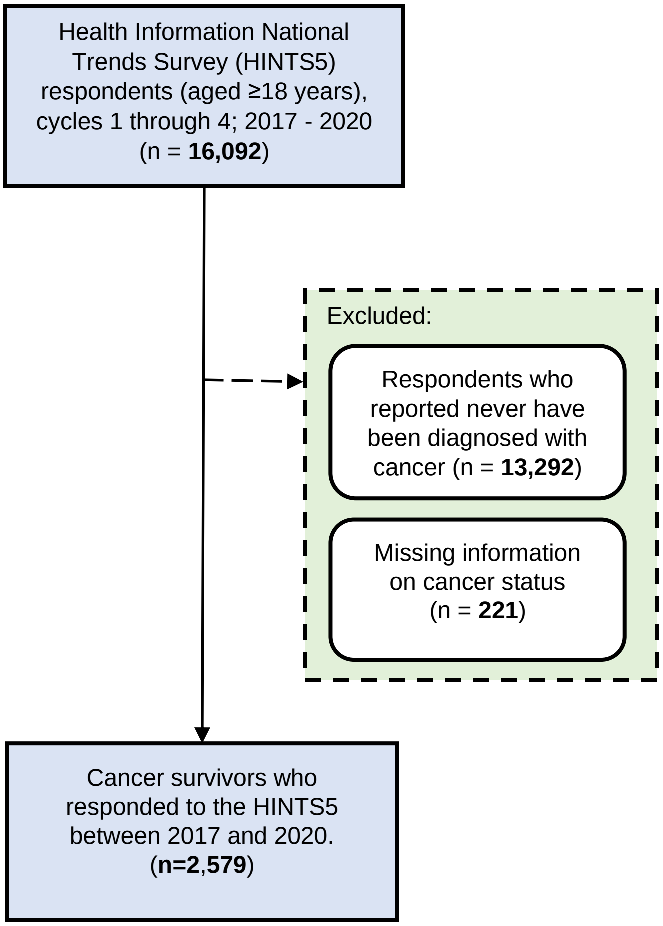

e-Figure 1. A flowchart depicting the study's inclusion and exclusion criteria.

e-Methods. Sensitivity analysis.

Sensitivity analyses were performed to check for the robustness and validity of the original findings. First, we excluded HINTS5-Cycle 4 responses for the surveys fielded after the global pandemic declaration of COVID-19 (i.e., March 11, 2020, onward, involving 359 responses). COVID-19 substantially impacted caregivers' and cancer survivors' mental health and well-being [1–5]. Also, it reshaped the caregiving paradigm with many individuals picking up caregiving responsibilities to care for family and loved ones in need of care during the pandemic. Second, all cycles of HINTS 5 involved a single-mode self-administered mail-in questionnaire. However, cycle 3 (fielded in 2019), was composed of a multimode survey and had two experimental conditions of a web pilot option in addition to the traditional mail-in questionnaire. Because mail-in and web-based surveys have inherent differences, their responses may vary across respondents' sociodemographic characteristics as are influenced by the existing digital divide [6–8]. Therefore, we excluded HINTS 5-Cycle 3 Web-only surveys fielded in 2019 (responses from 295 cancer survivors) and re-fit the adjusted models. The results of the sensitivity analyses are presented in e-Table 1 below.

e-Table 1. Findings of the sensitivity analyses performed for the associations between caregiving responsibility and levels of psychological distress among cancer survivors (HINTS5, Cycles 1 through 4, 2017–2020, the United States).

	Psychological distress	
	Mild vs. Normal	Moderate to severe vs. Normal
Caregiver (ref: no)	Adjusted OR (95% CI)	Adjusted OR (95% CI)
Original results	2.25 (1.17, 4.29)[a]	2.18 (1.07, 4.46)[a]
Sensitivity 1*	2.69 (1.32, 5.49)[b]	2.08 (1.05, 4.74)[a]
Sensitivity 2**	2.06 1.02, 4.20)[a]	2.05 (1.09, 4.52)[a]

Abbreviations: OR, Odds Ratio; CI, Confidence Interval.

a) *P*-value <.05; b) *P*-value <.01.

*Excluding HINTS5-Cycle 4 survey responses for the period after COVID-19 was declared a global pandemic (i.e., on or after March/11/2020) (excluded n=359).

**Excluding HINTS 5, Cycle 3 Web survey respondents (fielded 2019) (excluded n=295).

e-References

1. Ervin J, Fleitas Alfonzo L, Taouk Y, et al (2024) Unpaid caregiving and mental health during the COVID-19 pandemic—A systematic review of the quantitative literature. Plos one 19:e0297097

2. Dellafiore F, Arrigoni C, Nania T, et al (2022) The impact of the COVID-19 pandemic on family caregivers' mental health: a rapid systematic review of the current evidence. Acta Bio Medica: Atenei Parmensis 93:

3. Bailey C, Guo P, MacArtney J, et al (2022) The experiences of informal carers during the COVID-19 pandemic: a qualitative systematic review. International Journal of Environmental Research and Public Health 19:13455

4. Ayubi E, Bashirian S, Khazaei S (2021) Depression and Anxiety Among Patients with Cancer During COVID-19 Pandemic: A Systematic Review and Meta-analysis. J Gastrointest Canc 52:499–507. https://doi.org/10.1007/s12029-021-00643-9

5. Legge H, Toohey K, Kavanagh PS, Paterson C (2023) The unmet supportive care needs of people affected by cancer during the COVID-19 pandemic: an integrative review. J Cancer Surviv 17:1036–1056. https://doi.org/10.1007/s11764-022-01275-z

6. Converse PD, Wolfe EW, Xiaoting Huang, Oswald FL (2008) Response Rates for Mixed-Mode Surveys Using Mail and E-mail/Web. American Journal of Evaluation 29:99–107. https://doi.org/10.1177/1098214007313228

7. Kwak N, Radler B (2002) A comparison between mail and web surveys: Response pattern, respondent profile, and data quality. Journal of official statistics 18:257

8. Shih T-H, Fan X (2009) Comparing response rates in e-mail and paper surveys: A meta-analysis. Educational research review 4:26–40
